# Plasma virome of 781 Brazilians with unexplained symptoms of arbovirus infection include a novel parvovirus and densovirus

**DOI:** 10.1371/journal.pone.0229993

**Published:** 2020-03-05

**Authors:** Elizabeth Fahsbender, Antonio Charlys da-Costa, Danielle Elise Gill, Flavio Augusto de Padua Milagres, Rafael Brustulin, Fred Julio Costa Monteiro, Marlisson Octavio da Silva Rego, Edcelha Soares D’Athaide Ribeiro, Ester Cerdeira Sabino, Eric Delwart

**Affiliations:** 1 Vitalant Research Institute, San Francisco, CA, United States of America; 2 UCSF Dept. of Laboratory Medicine, University of California–San Francisco, San Francisco, CA, United States of America; 3 School of Medicine & Institute of Tropical Medicine, University of Sao Paulo, Infectious Disease, Sao Paulo, Brazil; 4 Public Health Laboratory State (LACEN/TO), Secretary of Health of Tocantins, Palmas, TO, Brazil; 5 Federal University of Tocantins, Palmas, Tocantins, Brazil; 6 Public Health Laboratory State (LACEN/AP), Secretary of Health of Amapa, Macapa, AP, Brazil; University of Kansas Medical Center, UNITED STATES

## Abstract

Plasma from patients with dengue-like symptoms was collected in 2013 to 2016 from the Brazilian states of Tocantins and Amapa. 781 samples testing negative for IgM against Dengue, Zika, and Chikungunya viruses and for flaviviruses, alphaviruses and enteroviruses RNA using RT-PCRs were analyzed using viral metagenomics. Viral particles-associated nucleic acids were enriched, randomly amplified, and deep sequenced in 102 mini-pools generating over 2 billion reads. Sequence data was analyzed for the presence of known and novel eukaryotic viral reads. Anelloviruses were detected in 80%, human pegivirus 1 in 19%, and parvovirus B19 in 17% of plasma pools. HIV and enteroviruses were detected in two pools each. Previously uncharacterized viral genomes were also identified, and their presence in single plasma samples confirmed by PCR. Chapparvovirus and ambidensovirus genomes, both in the *Parvoviridae* family, were partially characterized showing 33% and 34% identity in their NS1 sequences to their closest relative. Molecular surveillance using pre-existing plasma from febrile patients provides a readily scalable approach for the detection of novel, potentially emerging, viruses.

## Introduction

Deep sequencing of nucleic acids in clinical samples now allows the identification of any known infectious agents, resulting in improved diagnostic capabilities [1]. Analyzing plasma from patients with symptoms of acute viral infections such as fever of unknown origin may also be used as a surveillance tool for unexpected or novel (previously uncharacterized) viruses. Plasma or serum from 25 US men [2], 123 Nicaraguans [3], 23 US children [4], 498 Kenyans [5], 195 Nigerians [6], 90 Ugandans [7], 88 Peruvians [8], 12 and 135 Tanzanians [9, 10], and 40 UK resident returning from travels [11], all with unexplained fever or other signs of viral infections have shown the presence of nucleic acids from numerous human viruses. These viruses consist of both commensals and pathogens including anelloviruses, pegiviruses, papillomaviruses, hepatitis A and E, HHV-4/5/6/7/8, rhinovirus C, Merkel cell polyomavirus, HIV, HBV, HCV, norovirus, rotavirus, Dengue virus, West Nile virus, Chikungunya virus, mumps virus, astrovirus MLB1 and MLB2, adenovirus, and lassa virus.

Metagenomic analyses of plasma from patients with unexplained fever have also led to the characterization of previously unknown viral genomes. These genomes include an orthobunyavirus (Nyangole virus) from a Ugandan [7], a novel phlebovirus from a Tanzanian [12] and a rhabdovirus named Bas-Congo virus from a small hemorrhagic fever outbreak in the Democratic Republic of Congo [13]. Other rhabdoviruses named Ekpoma 1 and 2 were identified from the plasma of healthy West-Africans [6]. Virome analyses of blood donors with unexplained elevated liver enzyme markers have also been tested but did not identify novel human viruses [14, 15]. Blood derived DNA sequences generated during human genome studies [16] or pre-natal screen of pregnant women [17] have also been analyzed for viral DNA sequences.

Blood from healthy people with high exposure to viral infections has also been analyzed using metagenomics. Frequent transfusion recipients revealed a novel human virus named pegivirus 2 whose detection was associated with co-infection by HCV [18, 19]. French blood donors with positive markers for known blood-borne viruses such as HIV, HCV, or HCV as well as frequent transfusion recipients showed only the presence of known human viruses [20]. Lastly human plasma pools and blood-derived products can be similarly analyzed. Human plasma pools used for fractionation of biological products [21], Swiss platelet concentrates [22], and red blood cells and plasma units for transfusion [23], showed the presence of already known human viruses as well as small circular DNA viral genomes of unknown origin [21].

Here we describe a viral metagenomic analysis of plasma from 781 febrile Brazilian patients suspected of being infected with Dengue virus (DENV) based on their symptoms, but were negative for arbovirus RNA and anti-arbovirus IgM as well as enterovirus RNA. We report here on the known human viral genomes detected as well as two previously uncharacterized genomes, namely those of a chapparvovirus (the first reported in a human sample) and an ambidensovirus, both in the *Parvoviridae* family.

## Materials and methods

### Ethical review

Sample collections were approved by Institutional Research and Ethics committees and all participants or their guardians provided written informed consent prior to blood collection. Ethics Committee approval was granted by Faculdade de Medicina da Universidade de São Paulo (CAAE: 53153916.7.0000.0065), and Centro Universitário Luterano de Palmas ― ULBRA (CAAE: 53153916.7.3007.5516). Samples were collected by the central laboratories of public health (Laboratório Central de Saúde Pública or LACEN) of the states of Tocantins and Amapa. Samples were tested if patients showed three or more of the following symptoms: high fever that lasts for two to seven days, severe pain in the muscles, bones, and joints, pain behind the eyes, severe headaches, nausea and vomiting, rash, decrease in the number of white blood cells and a low level of platelets in the blood, and/or skin hemorrhages (bleeding under the surface of the skin) that appear as red or purple spots on the body.

### Testing for recent arbovirus and enterovirus infections

Detection of early anti-arbovirus immune responses were tested for using anti-Chikungunya Virus ELISA IgM assay (Euroimmun Medizinische Labordiagnostika AG, Luebeck, Germany), Anti-Zika Virus ELISA IgM assay (Euroimmun Medizinische Labordiagnostika AG, Luebeck, Germany) and the Panbio DENV NS1 antigen ELISA tests (Abbott, Chicago, IL, USA) according to each manufacturer's instructions using an ELISA reader (Bio-Rad, Hercules, USA) with absorbance at 450nm. For detection of viral RNAs a ZDC (Zika, Dengue, Chikungunya viruses) multiplex qPCR assay (Bio-Rad Laboratories, Inc.; Hercules, California, USA), was performed according to the manufacturer’s protocol. Negative samples were then tested using a multiplex pan-Flavivirus qPCR assay [24]. Samples negative for pan-flavivirus PCR were then submitted to a pan-Alphavirus multiplex qPCR assay [25]. The samples that showed negative results for the pan-Alphavirus assay were then submitted to a pan-Enterovirus real time PCR assay targeting the 5’ UTR [26].

### Metagenomic analysis

Plasma samples were pooled based on location in groups of 4 to 10 samples. Viral particle-associated nucleic acids were enriched by filtration through a 0.45 μm filter (Millipore, Burlington, MA, USA) and filtrate digested for 1.5 hours at 37°C with a mixture of nuclease enzymes consisting of 14U of Turbo DNase (Ambion, Life Technologies, USA), 3U of Baseline-ZERO (Epicentre, USA), 30U of Benzonase (Novagen, Germany) and 30U of RNase One (Promega, USA) in 1x DNase buffer (Ambion, Life Technologies, USA) to reduce the content of non-encapsidated human nucleic acids. Nucleic acids were then extracted using the MagMAX^TM^ Viral RNA Isolation kit (Applied Biosystems, Life Technologies, USA). Nucleic acids were incubated for 2 min at 72⁰C with 100 pmol of primer A (5’GTTTCCCACTGGANNNNNNNN3’) followed by a reverse transcription step using Superscript III (Invitrogen) with a subsequent Klenow DNA polymerase step (New England Biolabs). cDNA was then amplified by a PCR step using AmpliTaq Gold^™^ DNA polymerase LD with primer A-short (5’GTTTCCCACTGGATA3’). The randomly amplified products were then quantified by Quant-iT^™^ DNA HS Assay Kit (Invitrogen, USA) using Qubit fluorometer (Invitrogen, USA). 1ng of DNA from each pool was then converted to Illumina sequencing compatible DNA using the transposon-based Nextera kit with 15 PCR cycles [27]. Equimolar products were then pooled and sequenced using 3 lanes of HiSeq 4000 paired end 150 bases yielding a total of 2,060,293,312 reads with a median of 19,558,831 and an average of 20,198,954 reads per pool with a wide range of 7,622 to 57,630,090 reads per pools. The raw data has been submitted to the short read archive under PRJNA602336.

### Bioinformatics

Duplicate and low-quality reads were removed and the Ensemble program [28] was used to assemble contigs. Both contigs and singlets were then analyzed using BLASTx (v.2.2.7) to all annotated viral proteins sequences available in RefSeq of GenBank. To account for index hopping, a threshold of greater 10 reads per million was set for samples that shared indices with the samples with the highest read numbers. If the pool had 10 reads per million or less of anelloviruses, parvovirus B19, and pegivirus, the reads were removed from analysis. The short read sequencing data is available at NCBI Sequence Read Archive (SRA) under the BioProject number PRJNA602336.

### Chapparvovirus and ambidensovirus PCR detection

A nested PCR was used targeting the NS1 gene of the human-associtaed chapparvovirus with first-round primers: Hchappa_F (5’-GTCGGTAGGGTCTTGATGCA-3’) and Hchappa_R (5’- CGGCTGCGCTGTTGTTAAAA-3’) to amplify a 396-nt product, and second round primers Hchappa_FN (5’-TGGGGCGAAATACAAAGCAG-3’) and Hchappa_RN (5’- CCAATCAGAGGCTCTTCCCA-3’) to amplify a 85-nt second-round product. Ambidensovirus DNA was detected using a nested PCR targeting the NS gene with first round primers Hambi_F (5’- CGACTGCACCAACAATTGCA-3’) and Hambi_R (5’- TTCTTCGAGCGTACGCAGAG-3’) amplifying a 568–nt product, followed by a second PCR round with Hambi_FN (5’- CCACGGGATCCAAATGTCCA-3’) and Hambi_RN (5’- ATAGCGAACGCAGTAGAGGC

-3’) amplifying a 200-nt product. PCRs contained a final concentration of 0.2 μm of each primer, 0.2 mM of dNTPs, 0.625 U of Amplitaq Gold^®^ DNA polymerase (Applied Biosystems, Waltham, MA, USA), 1× PCR Gold buffer II, 1.5 mM of MgCl_2_ and 1 μL of DNA template in a 25 μl reaction) cycled as follows: 95°C for 5 min, 40 cycles of 95°C for 30 s, then 52°C for the first round and 54°C for the second round of PCR for 30 s, and 72°C for 30 s followed by a final extension at 72°C for 7 min. PCR products of the correct size were identified by gel electrophoresis and confirmed by Sanger sequencing.

### Phylogenetic analysis

The amino acid sequences of the NS1 were aligned using MUSCLE and a Maximum likelihood tree was created using the Jones–Taylor–Thorton matrix-based model with 1,000 bootstrap replicates in MEGA6.0 [29–31].

## Results

Plasma samples collected in 2013–2016 from patients with suspected cases of arbovirus infections were provided by the Central Laboratories of Public Health of Tocantins and Amapa states (Laboratório Central de Saúde Pública or LACEN). Samples testing negative for recent infections by Dengue, Zika, or Chikungunya viruses, using IgM tests and negative for viral RNA using pan-Flaviruses, pan-Alphaviruses, and pan-Enteroviruses RT-PCR assays (see Materials and Methods) were selected for viral metagenomics analyses. Samples from Tocantins were collected in 2016 while those for Amapa residents were collected over 4 years (2013–2016). The age distribution and gender of patients from Tocantins and Amapa states are shown in Table 1. The average age was 27 for Tocantin and 29 for Amapa patients.

**Table 1:** Age range and gender of patient samples.

**Tocantins**					
	Number of patients			Average age	
age range	total	male	female	male	female
0–9	92	26	66	3	3
10–19	30	18	12	15	15
20–29	47	29	18	23	25
30–39	47	21	26	33	34
40–49	47	15	32	43	43
50–59	33	17	16	54	53
60–69	13	6	7	63	64
70–79	3	2	1	72	71
80–89	2	2	0	86	n/a
Total	314	136	178	-	-
**Amapa**					
	Number of patients			Average age	
age range	total	male	female	male	female
0–9	79	48	31	5	5
10–19	85	51	34	14	15
20–29	68	31	37	25	24
30–39	104	46	58	34	34
40–49	60	31	29	44	44
50–59	37	12	25	54	54
60–69	23	8	15	63	62
70–79	11	7	4	73	73
80–89	-	-	-	-	-
Total	467	234	233	-	-

### Virome characterization

**Fig 1:**
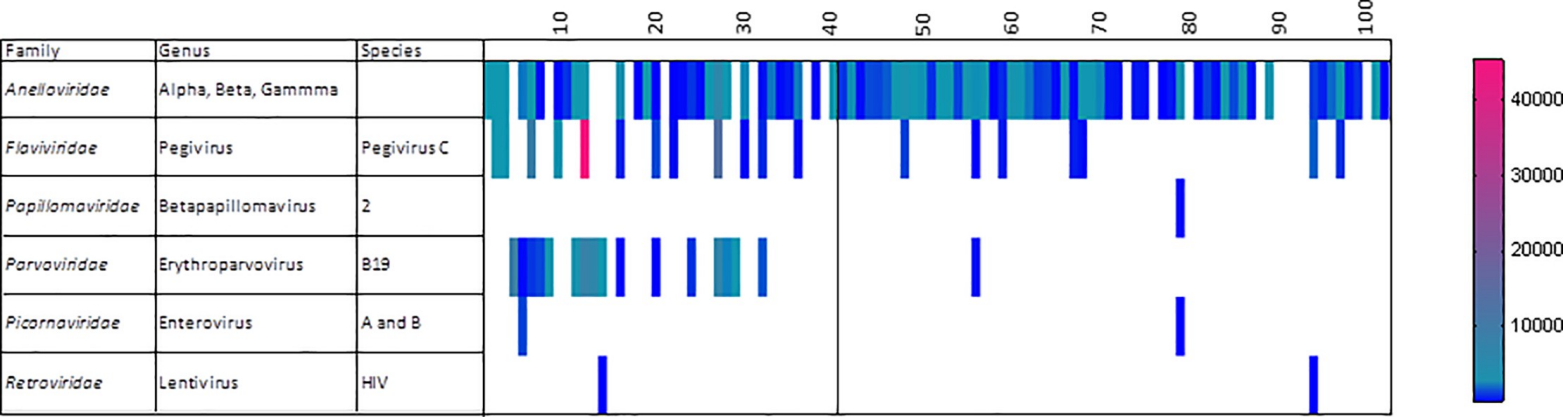
Heat map of human viral reads per million total reads.

Plasma samples were then pooled in groups of 4 to 10 samples from the same year and state and processed to enrich for viral particle-associated nucleic acids (Materials and Methods). The resulting sequence data was then analyzed for high sequence similarity to known human viruses using an E score cut-off of E<10^−10^ (Fig 1). Viral sequences detected in the largest fraction of plasma pools belonged to the *Anelloviridae* family and were found in 82/102 (80%) of pools. Next in frequency were sequences belonging to the *Pegivirus* genus (Pegivirus C species aka GBV-C or human pegivirus 1 in the *Flaviviridae* family) found in 19/102 pools (19%). Reads matching the recently described pegivirus H species (hepegivirus aka human pegivirus 2, NC_038436)[18, 19] were not detected. Next in prevalence was protoparvovirus B19 in the *Parvoviridae* (genus *Erythroparvovirus*) family. The prevalence of B19 sequence detection was higher in the plasma pools from Tocantins (16/41 pools positive) than in those from Amapa (1/61 pools positive). The average age of the sampled patients from these two states was 27 for Tocantins and 29 for Amapa.

More sporadic detection of human viruses consisted on a single pool containing human papillomavirus genotype 100 in the *Betapapillomavirus* 2 species (GB FM955839.1) reported in cutaneous tissues as well as mucosal lesions [32]. Two different picornaviruses, Coxsackievirus A16 and Echovirus 30, were both found in the same Tocantins pool while Coxsackievirus A5 was found in an Amapa pool. HIV1 was found in two pools (both HIV1M subtype B).

### Newly characterized viruses

**Fig 2:**
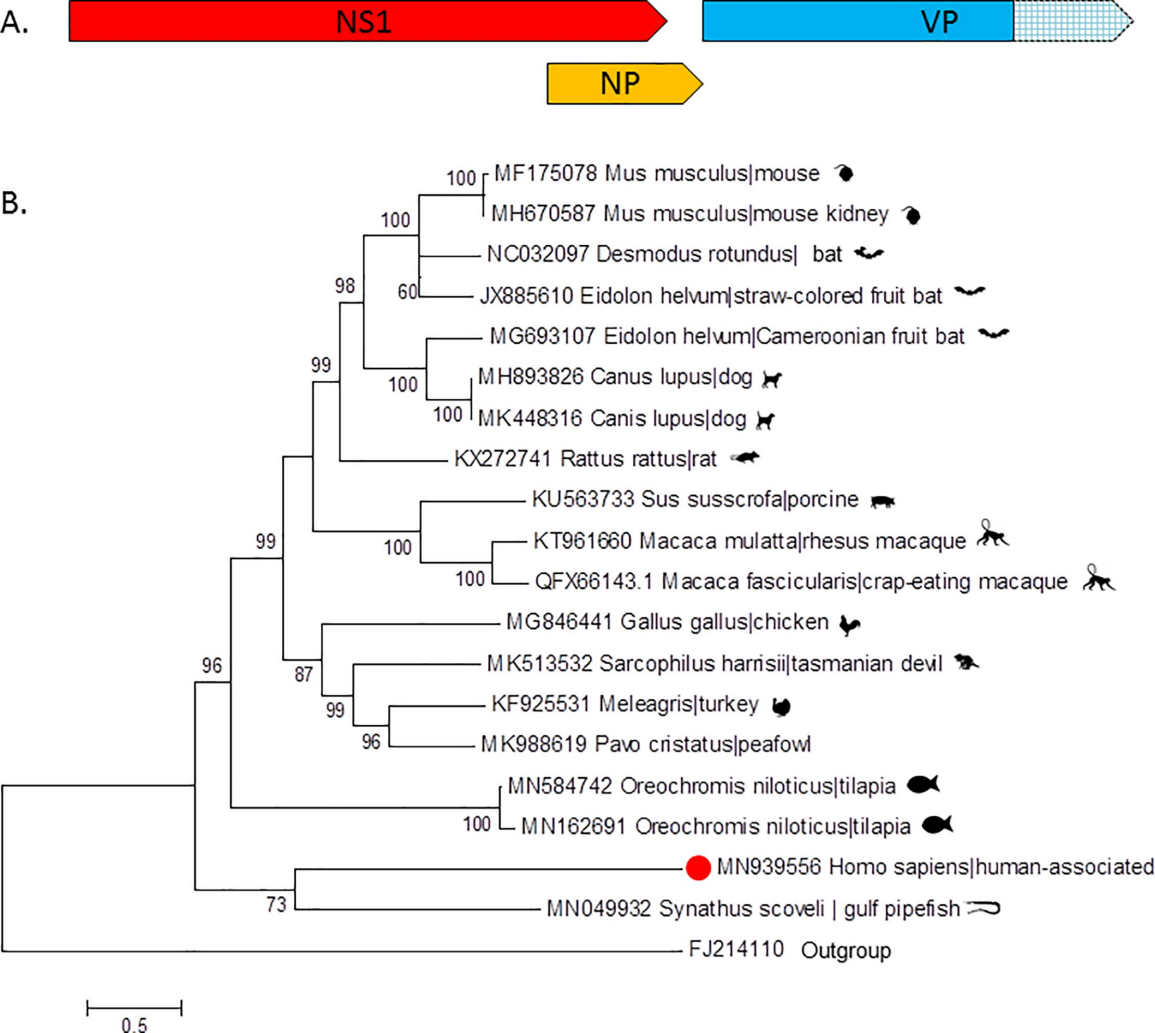
A. Genome schematic of human-associated chapparvovirus. Hatched box represent section of VP gene that was not sequenced. The complete non-structural (NS1) protein ORF (656aa) is in red, the NP is in orange (347 aa), and the partial VP in in blue. B. Maximum likelihood trees of chapparvovirus NS1 aa sequences. Bar, 0.5 amino acid substitutions per site. Bootstrap values below 60 were removed.

**Fig 3:**
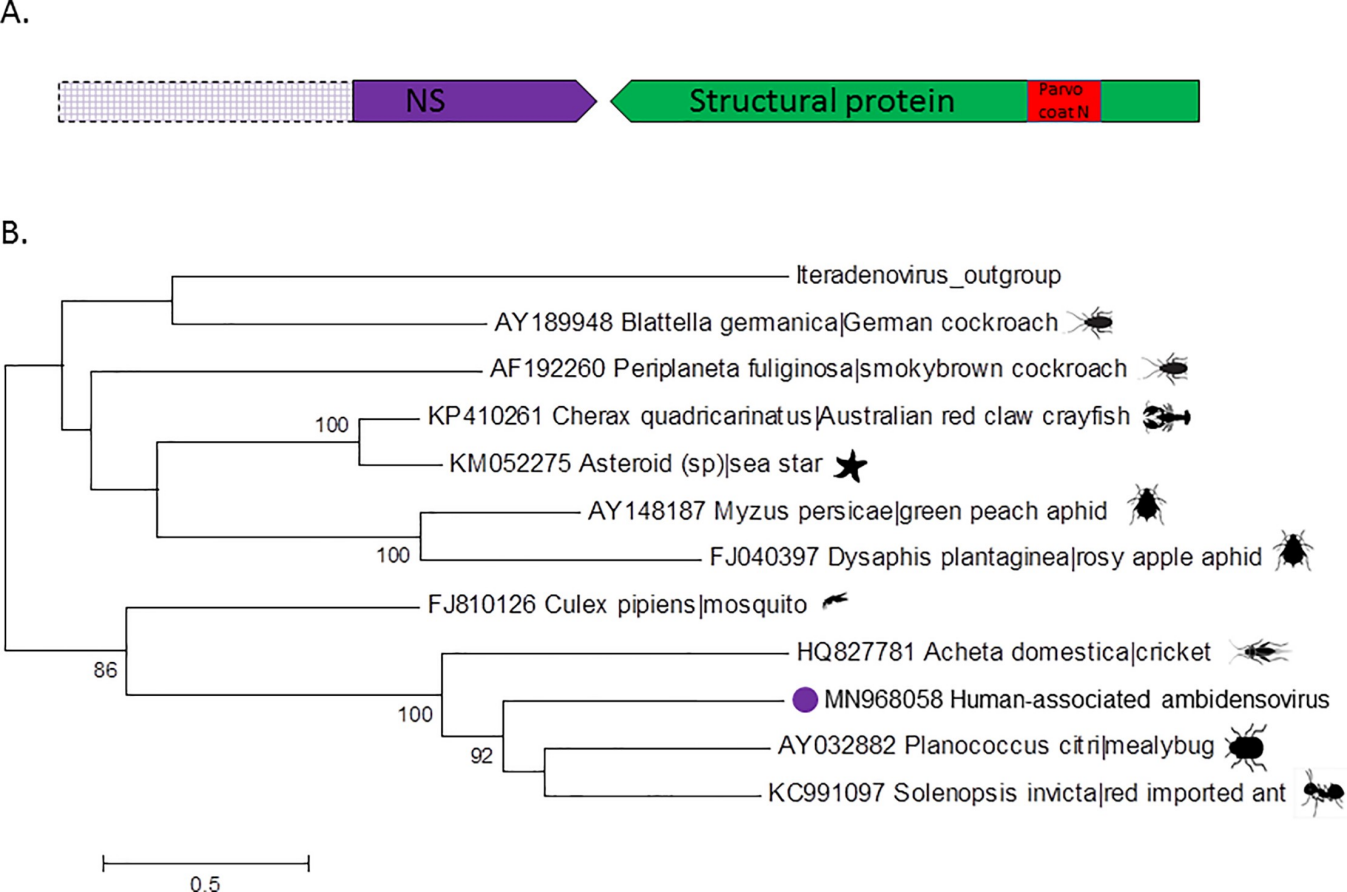
A. Genome schematic of human-associated ambidensovirus. Hatched boxes represent section of NS that were not sequenced. The purple ORF represents the partial non-structural (NS) protein, the green ORF encodes the complete structural protein (530 aa), and the red represents the conserved domain of the parvovirus VP coat protein. B. Maximum likelihood trees of partial NS1 aa sequences. Bar 0.5 amino acid substitutions per site. Bootstrap values below 60 were removed.

Also detected in two pools were sequences with more limited similarity to proteins of eukaryotic viruses, namely of chapparvoviruses and ambidensoviruses both (like protoparvovirus B19) ssDNA viruses part of the *Parvoviridae* family. Ambidensoviruses are classified in the *Densovirinae* subfamily while chapparvoviruses are classified in a newly formed third *Parvoviridae* subfamily named *Hamaparvovirinae*. These viral reads were identified with BLASTx E scores ranging from 0.007 to 10^−6^ (for chapparvovirus singlet reads) and 0.002 to 10^−11^ (for ambidensovirus singlet reads) depending on the protein region compared. Contigs were also generated following de novo assembly with length of 2405 bases for the chapparvovirus and 1421 to 3196 bases for the ambidensovirus resulting in better BLASTx E scores of 10^−31^ and 10^−49^ to 10^−66^ respectively. The individual plasma sample within each of the two pools that initially yielded the new viral DNA sequences were each extracted and a single virus-containing sample identified by nPCR using primers based on the short Illumina reads (Materials and Methods). Each of these two individual plasma samples was then processed as described for the pools to enrich for viral particles and sequenced using an Illumina MiSeq in order to sequence more of the viral genomes (Materials and Methods). Sequencing of the chapparvovirus yielded a complete NS ORF and a partial VP sequence (Fig 2A), while the ambidensovirus yielded a partial NS and complete VP sequence (Fig 3A). No other human viruses were detected in these two individual plasma samples beside anelloviruses. Further attempt to complete the 3’ region of the chapparvovirus using 3’ RACE were unsuccessful. In order to determine whether these viral sequences were present in other pools the random-PCR products from each of the 102 libraries were screened using nested PCRs targeting both viruses (Materials and Methods). No other pools were positive. Chapparvovirus DNA was therefore detected in a single patient from the state of Tocantins and ambidensovirus DNA in a single patient from the state of Amapa.

The NS sequences were then used for phylogenetic analyses using the closest relatives available in GenBank. The closest relative to the human-associated chapparvovirus (MN939556) NS sequence was from a Gulf pipefish but the large genetic distance between the two viruses (33.3% identity over 56% of NS) argues against any recent cross-species transmission (Fig 2B). The best match for the partial VP sequence was to the Tasmanian devil-associated chapparvovirus 1 (36.8% identity of 85% of partial VP protein). A basal position of the human-associated and Gulf-pipefish chapparvoviruses NS sequences, relative to the other vertebrate-associated chapparvoviruses, was observed (Fig 2B). The large genetic distance between these two chapparvoviruses argues against a recent common ancestry.

For the ambidensovirus (MN968058) the closest NS sequence belonged to a virus from *Solenopsis invicta* (Red imported fire ant) (Fig 3B) (a member of the proposed *Scindoambidensovirus* genus within the *Densovirinae* subfamily) [33] but as for the chapparvovirus the large genetic distance between these proteins argues also argues against recent common ancestry.

## Discussion

We describe here the plasma virome of febrile Brazilian testing negative for Dengue RNA. We detected typical members of the human plasma virome, namely anelloviruses and human pegivirus 1. These two viruses are highly prevalent world-wide and generally considered commensal infections, inducing no significant pathology [34, 35]. While human pegivirus 1 reads were found in 18% of pools no reads matching human pegivirus 2 (HPgV2) were detected. The presence of HPgV2 has been often [18, 19, 36–39] although not exclusively [40] linked with co-infection by HCV which were not detected in this study.

Frequently detected and a possible source of febrile illness were parvovirus B19 seen in 17% of the tested plasma pools. B19 DNA was also commonly found in plasma pools of Kenyan adults with symptoms of primary HIV infection but HIV RNA negative [5]. Parvovirus B19 DNA was also recently reported in a large fraction of Brazilian adults with Dengue-like symptoms [41]. B19 viremia may therefore account for a significant fraction of unexplained fever in Brazilian adults. HIV and enterovirus RNA viremia were also detected in 2 pools each possibly accounting for a small fraction of patients with Dengue-like symptoms. The detection of enterovirus reads despite negative pan-enterovirus RT-PCR results may be due to lower sensitivity of this pan-PCR and/or the presence of mutations in the PCR primer binding sites.

Two novel viral genomes were also detected, their presence in unique plasma samples confirmed by PCR, and their genome partially characterized. These viruses belong either to a viral clade known to infect vertebrates but not previously reported in human (*chapparvovirus*) or known to infect invertebrates but not vertebrate hosts (*ambidensovirus*). Diverse chapparvovirus genomes, fecally shed by non-human mammals, including rhesus macaques [42] and cynomologus macaques [43], rats and mice [44, 45], bats [46–48], pigs [49], Tasmanian devils [50], dogs [51], birds (including turkey [52], red-crowned crane [53], chicken [54] and peafowls [55]) and in fish (tilapia fish [56] and Gulf pipefish [33]) have recently been described. A murine chapparvovirus, initially described in wild New York City mice feces [45], was independently re-discovered and shown to be kidney-tropic and pathogenic to laboratory mice [57]. These prior reports of chapparvoviruses in numerous mammals including non-human primates make the novel chapparvovirus described here a plausible human-infecting virus.

The detection of a nuclease-resistant densovirus genome in human plasma was more surprising. Densoviruses can infect a wide range of invertebrates including many insects as well as crustaceans and echinoderms such as starfish and sea urchins [58, 59]. Some densoviruses have been used to inoculate vertebrate cells unsuccessfully [60, 61]. In contrast a densovirus genome was detected by metagenomics and PCR confirmed in a human cerebral spinal fluid from an unexplained case of encephalitis [62]. Another densovirus was detected by PCR in the lung tissue of a bird (*Parus major* or great tit) and its presence also confirmed by re-extraction and PCR. Inoculation of feline kidney (F81) cells with this avian lung tissue resulted in cytopathic effects with hypertrophied nuclei typical of insect cells infected with densoviruses [63]. Genomes from other viral families currently only known to infect non-vertebrate hosts such as dicistroviruses (insects) and partitiviruses (fungi) have also been described in human plasma [8, 64]. Genomes from a group of viruses with circular Rep encoding ssDNA (CRESS-DNA) genomes named gemycircularvirus, recently classified in a diverse viral family named *Genomoviridae* [65], have been reported in human plasma pools [21] and other human plasma samples [66–68]. To date the cellular tropism of only a single gemycircularvirus has been determined consisting of both a fungi [69] and fungus-eating insect [70]. Detection of such viral nucleic acid in human plasma may conceivably reflects expanded viral tropism of a subset of otherwise largely invertebrate or fungi tropic viral groups to include human cells resulting in a viremia sufficiently high for detection using metagenomics. Alterative explanation also exist such as parasitic infections with fungi, protozoa, nematodes, and/or insects and concomitant release of their viruses into the human blood stream or diffusion of ingested viruses from the gut into the blood. Some form of contamination, which continues to plague the field of viral discovery, [71–74] may also account for these unexpected viral nucleic acids detections in normally sterile human plasma. Confirmation using PCR or RT-PCR that the original biological samples indeed contained novel viral genomes was used here to exclude contamination occurring during generation of metagenomics libraries [62, 75, 76]. PCR detection in individual, newly re-extracted plasma samples, using different reagents from those used for the construction of the metagenomic libraries, indicated that their initial detection by deep sequencing was not the result of late stage contamination. Contaminations occurring earlier such as from air-borne particles drifting into open tubes or from contaminated skin during phlebotomy cannot be excluded. Indeed a beta-papillomavirus was detected here and Merkel cell polyomavirus (a common skin tropic virus)[77] has been frequently reported in blood samples [5, 8, 16, 23, 78, 79].

Further testing the human tropism of the human plasma-associated parvovirus and densovirus reported here will require detection of specific antibody responses, viral amplification in human cells, and/or the detection of viral RNA in cells of infected tissues. The availability of these genomes in GenBank will also facilitate their future detection through metagenomics or PCR studies to better define their possible association with human symptoms and presence in animal reservoirs.
